# Mice Against Ticks: an experimental community-guided effort to prevent tick-borne disease by altering the shared environment

**DOI:** 10.1098/rstb.2018.0105

**Published:** 2019-03-25

**Authors:** Joanna Buchthal, Sam Weiss Evans, Jeantine Lunshof, Sam R. Telford, Kevin M. Esvelt

**Affiliations:** 1 MIT Media Lab, Massachusetts Institute of Technology, Cambridge, MA 02142, USA; 2 Program on Emerging Technology, Massachusetts Institute of Technology, Cambridge, MA 02155, USA; 3 Program on Science, Technology, and Society, Tufts University, Medford, MA 02138, USA; 4 Program on Science, Technology and Society, Kennedy School of Government, Harvard University, Cambridge, MA 02142, USA; 5 Department of Genetics, Harvard Medical School, Boston, MA 02115, USA; 6 Department of Genetics, University Medical Center Groningen, University of Groningen, 9700 RB Groningen, The Netherlands; 7 Department of Infectious Disease and Global Health, Cummings School of Veterinary Medicine, Tufts University, N. Grafton, MA 01536, USA

**Keywords:** ticks, Lyme disease, heritable immunization, ecological engineering, community-guided, CRISPR

## Abstract

Mice Against Ticks is a community-guided ecological engineering project that aims to prevent tick-borne disease by using CRISPR-based genome editing to heritably immunize the white-footed mice (*Peromyscus leucopus*) responsible for infecting many ticks in eastern North America. Introducing antibody-encoding resistance alleles into the local mouse population is anticipated to disrupt the disease transmission cycle for decades. Technology development is shaped by engagement with community members and visitors to the islands of Nantucket and Martha's Vineyard, including decisions at project inception about which types of disease resistance to pursue. This engagement process has prompted the researchers to use only white-footed mouse DNA if possible, meaning the current project will not involve gene drive. Instead, engineered mice would be released in the spring when the natural population is low, a plan unlikely to increase total numbers above the normal maximum in autumn. Community members are continually asked to share their suggestions and concerns, a process that has already identified potential ecological consequences unanticipated by the research team that will likely affect implementation. As an early example of CRISPR-based ecological engineering, Mice Against Ticks aims to start small and simple by working with island communities whose mouse populations can be lastingly immunized without gene drive.

This article is part of a discussion meeting issue ‘The ecology and evolution of prokaryotic CRISPR-Cas adaptive immune systems’.

## 1. Introduction

The prospect of using CRISPR to solve ecological problems by editing the genomes of wild populations has generated considerable interest [1,2]. Claims of applications with major potential benefits, some doubtless inflated but others with working laboratory examples [3], have sparked wide-ranging and sometimes contentious debates about the role of this area of science in society [4]. The resulting social, diplomatic and regulatory challenges may be more formidable than technical development.

Popular attention has focused on CRISPR-based gene drive systems, the most powerful of which may be capable of unilaterally editing entire wild populations of organisms. Mathematical models predict that ‘self-propagating’ CRISPR gene drives will spread to most populations of the target species that are connected by gene flow [5–7], a prediction supported by the observed spread of the natural P element gene drive to every wild population of the fruit fly *Drosophila melanogaster* on six continents during the middle half of the twentieth century [8]. Given the history of human-mediated transport of wild organisms and the media attention focused on gene drive, it is not unreasonable to assume that self-propagating CRISPR gene drive systems will eventually affect all populations of the target species.

**Figure 1.**
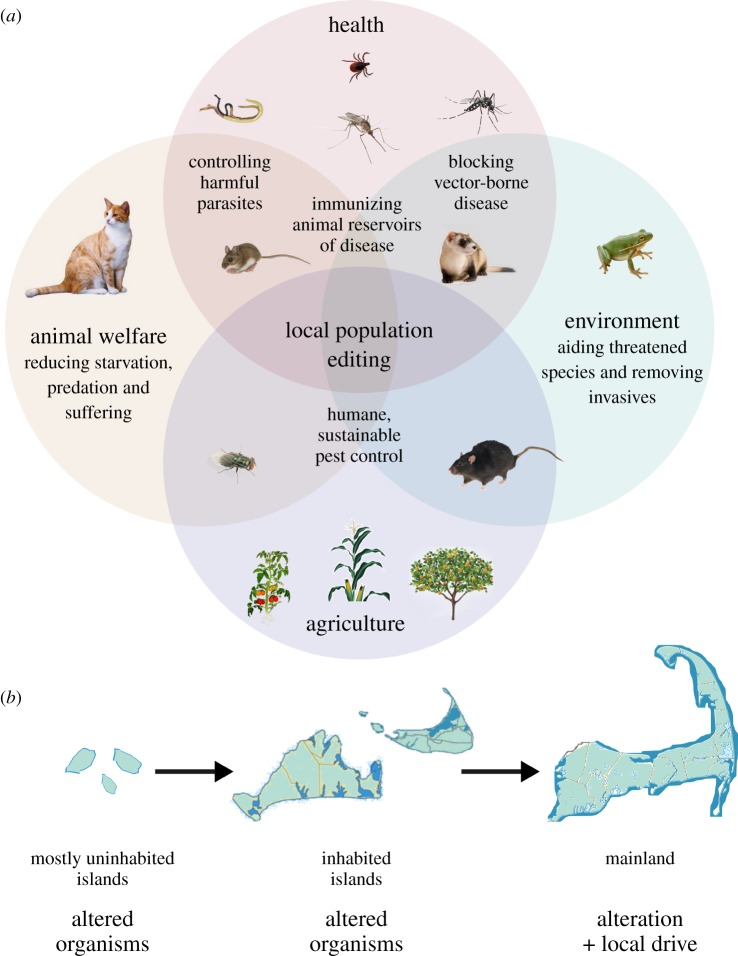
(*a*) The vast majority of environmental genome editing applications seek to alter a local population of the target species. (*b*) Because environmental effects are specific to the organism and alteration, the simplest test may involve releasing edited organisms on mostly uninhabited islands without any form of drive. This might be followed by inhabited island communities choosing to release organisms, and only then by adding a local drive system to enable mainland communities to spread and/or maintain the alteration there.

Yet very few proposed applications intend to affect an entire species, and even fewer are considered important enough to work towards an international agreement on use without a field trial of the technology. The *Anopheles gambiae* mosquitoes that are the primary vectors for malaria in Africa may cause enough harm to catalyse such an agreement [9]; the same might be true of the human schistosomes *Schistosoma haematobium* and *S. mansoni* and perhaps the New World screwworm *Cochliomyia hominivorax*. All other proposed applications of CRISPR-based genome editing in wild populations involve altering a specific local population (figure 1*a*).

The academic and popular media's focus on gene drive can easily distract from discussions about ecological effects of editing wild organisms. The approach discussed here, in contrast, focuses first on these ecological effects, only considering local gene drive as a potential method of introduction once the effects of mouse immunity are understood.

Alterations can be introduced to populations without any form of gene drive by simply releasing a sufficiently large number of organisms carrying the desired change. A strategy termed ‘inundative release’ has been classically used for population suppression by releasing multiple sterilized insects for each wild counterpart; a similar approach with fertile organisms can introduce engineered changes.

Islands are a special case: because there is little gene flow with larger wild populations that would otherwise dilute an introduced alteration, it is feasible to stably alter island populations by releasing edited animals without the complications of a drive. Because ecosystems are complex and not always well understood, it may be prudent to test the effects of specific alterations in isolation before adding the complication of a drive system. Initial field trials on mostly uninhabited islands might be followed by communities of larger, inhabited islands choosing to release edited animals (figure 1*b*). If successful, other island or mainland communities might then choose to add some form of local drive to alter their own populations.

Our emphasis on the role of communities is deliberate, as the application of genome editing to the shared environment is similar to infrastructure development and compulsory public health measures in creating public goods that can only be provided for some if provided for many. Because there is no possibility for residents to opt out of the effects, such issues can become divisive [10]. Deciding whether, when, and how to proceed are questions of civic governance rather than informed consent [2,11].

Once developed, ecotechnology ‘products’ are likely to be governance options analogous to public health measures such as iodine supplementation [12], which may in practice be deployed as commercial ventures with government oversight. But in the research phase, we believe that ecotechnology measures are more similar to non-commercial infrastructure development, where projects assume one of many possible forms as determined by key early stage decisions. Early stage infrastructure decisions, at least in democratic societies, have been found to be more successful with early community input [13]. The Mice Against Ticks project discussed here may serve as an example of what such involvement might look like during the development of CRISPR-based ecological engineering projects.

To date, the scientists involved in Mice Against Ticks (J.B, S.R.T, K.M.E) have sought to (1) understand the role ecological problems play in local communities, (2) describe a range of potentially feasible technical options, (3) engage with local community members to determine which (if any) to pursue, then (4) develop the technology according to community preferences. Here, we describe Mice Against Ticks as an experimental effort to iteratively engage members of the community in an effort to solve a public health problem by using CRISPR to edit wild animals that serve as reservoirs of disease.

## 2. Tick-borne disease

Lyme disease is the most frequently reported vector-borne illness in the USA, infecting over 300 000 Americans each year, the vast majority of whom live in the Northeast or Upper Midwest [14]. The causative spirochete bacterium (*Borrelia burgdorferi*) is transmitted by the black-legged (deer) tick *Ixodes scapularis,* the disease vector in eastern North America. This same tick also transmits the causative agents of babesiosis, anaplasmosis, ehrlichiosis and Powassan encephalitis. All except Powassan virus are treatable if caught early, but many cases are undiagnosed, often leading to lifelong complications such as Lyme arthritis, heart block and radiculitis [15].

**Figure 2.**
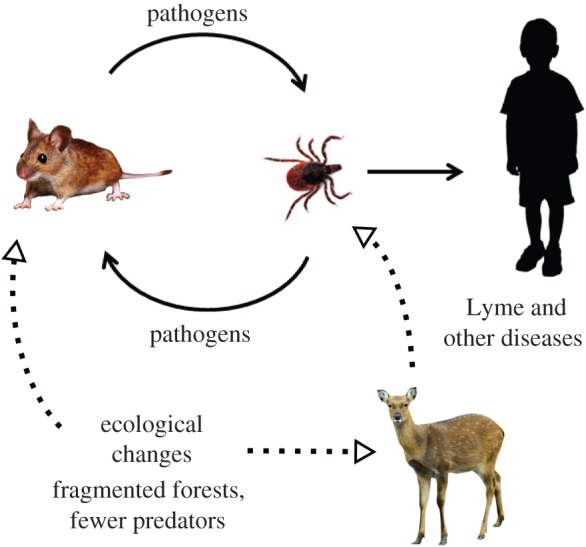
The white-footed mouse *Peromyscus leucopus* is an important reservoir of most pathogens transmitted by the deer tick *I. scapularis* owing to efficient bidirectional infection. Ecological changes have increased the number of deer, and therefore the number of ticks, as well as the abundance of white-footed mice, greatly increasing human infection rates. (Online version in colour.)

Tick-borne zoonoses are the result of spillover from an ecological cycle in which ticks infect mammalian hosts, which subsequently infect the next generation of ticks (figure 2) [16]. The white-footed mouse *Peromyscus leucopus* is an important reservoir for the pathogens transmitted by deer ticks. This mouse is easily infected by the spirochetal agent of Lyme disease as well as all of the other members of the deer tick microbial guild [17], efficiently serves as a source of infection for ticks and appears to feed a major proportion of subadult deer ticks in most northeastern US endemic sites. The density of the tick vector depends primarily on that of deer, which are the third and final (reproductive) host [18]. Over the past several decades, social and ecological changes such as fragmented reforestation and suburbanization have led to an explosion in the deer population, which has significantly increased the number of ticks [18], while also favouring white-footed mice [19,20]. The result has been a dramatic increase in the number of infected ticks and a correspondingly increased risk to people. Hence, epidemic tick-borne disease is an anthropogenic ecological problem.

The islands of Nantucket and Martha's Vineyard have some of the highest *per capita* rates of confirmed and probable Lyme disease cases in the USA [21]. According to the former Chairman of Nantucket's Board of Health, 40% of households have been directly affected (M Macnab 2016, personal communication). Both islands also have unusually high rates of babesiosis and other infections, especially Nantucket. Lyme disease and many of the deer tick microbial guild are also global health burdens from western Europe to Japan.

Many potential interventions exist to reduce the risk of Lyme disease or other tick-borne infections. One of us (S.R.T.) has been working closely with the communities of Nantucket and Martha's Vineyard on tick-borne disease prevention for 30 and 20 years, respectively, and has failed to persuade communities to commit to any intervention. Most may be classified as short-term approaches, in which resources and energy must be expended on a regular basis in perpetuity; once such actions are relaxed, risk returns to pre-intervention levels. Short-term interventions include personal protection (repellents, protective clothing, showers, tick checks), bait stations, oral vaccination of reservoir hosts and treating yards with acaricide barrier sprays [22].

No one-time intervention capable of long-lasting effects has been proposed previously. A vaccine against Lyme disease approved in 1998 for high-risk individuals aged 15–70 conferred 76% protection [23], but was voluntarily withdrawn by its manufacturer owing to declining sales [24]. Efforts to reintroduce this vaccine and to develop an alternative [25] are underway, but both are estimated to be perhaps a decade from market and may face strong opposition from anti-vaccination activists.

Deer reduction has been demonstrated to be effective in reducing tick densities in physically isolated sites such as islands and peninsulas, but unless local eradication is achieved, maintaining the benefits requires annual maintenance to keep the herd size to a target density. Sociopolitical factors including federal and state laws, private land ownership, opposition to firearms, desire from hunters to preserve hunting as a hobby and affection for deer tend to prevent routine adoption of this particular intervention even on islands where one-time eradication could be feasible. Other possible long-term approaches, such as deforestation, have yet to be explored. Integrated pest management can be highly effective, but economic, social and political challenges to implementation have combined with ecological changes such that the epidemic curve for Lyme disease continues to rise unabated.

## 3. Mice against ticks

Seeking applications that could set a precedent for open, community-guided ecotechnology development, one of us (K.M.E.) conceived of heritably immunizing wild white-footed mouse populations against tick-borne disease using antibodies derived from natural adaptive immunity. Crucially, introducing sufficient engineered resistance alleles into an island white-footed mouse population might reduce the reservoir competence of a key host for many decades or even centuries without requiring any form of gene drive. On an island, these introduced alleles could be subsequently removed by trapping animals and reintroducing wild-type organisms, offering a reversible way of assessing ecological effects.

If a simple version of this type of intervention were successful at preventing disease on offshore islands, more technically sophisticated localized gene drive systems [26] might enable mainland communities to subsequently immunize their own populations—an example of starting small and simple before scaling up. Even a partial reduction in the force of transmission in the natural cycle would likely provide a major public health benefit.

In consultation with colleagues with expertise in science and society (including S.W.E), we ran a direction-finding workshop in December 2015 at MIT to determine whether the approach was sufficiently promising to justify approaching the island communities of Nantucket and Martha's Vineyard, and if so, how best to do so. Attending were ecologists (including S.R.T.), molecular biologists (including K.M.E. and J.B.), medical doctors, science policy academics (including S.W.E.), ethicists (including J.L.), science educators, state and federal regulators, and representatives from island communities and environmental NGOs.

With respect to molecular biology, the proposed approaches to introduce heritably resistant mice were deemed technically feasible on the basis of gene therapy experiments in which mice given the ability to produce antibodies were protected against numerous pathogens [27–34]. Combined with advances in the use of CRISPR for germline editing, attendees concluded that conferring heritable resistance to tick-borne diseases would be a challenging engineering problem that would take years to accomplish, but attainable using current CRISPR editing techniques. Moreover, heritable resistance was determined to be more cost-effective at scale than less complex, alternative solutions like by-hand vaccination and bait-based vaccines, especially when considering options for mainland communities.

Past studies involving the vaccination of wild white-footed mice with recombinant OspA, a major *B. burgdorferi* protein that induced transmission-blocking immunity, indicated that even inefficiently immunizing an important reservoir can indeed substantially reduce local mouse and tick infection rates [35,36]. The attendees jointly concluded that the options were feasible and would likely be of interest to the communities of Nantucket and Martha's Vineyard, and that the research team should contact the islands' Boards of Health to schedule presentations for their board members and interested community members.

In June 2016, we (J.B., S.R.T. and K.M.E.) presented a variety of technical options to approximately 30 community members at a Nantucket Board of Health meeting. The following month, we gave the same presentation at a meeting of the Health Agents from the six towns of Martha's Vineyard and separately to a gathering of a 100 residents and island visitors at the Edgartown library. Videos of two of the three presentations and subsequent town meetings are publicly available on Responsive Science, a site dedicated to documenting and facilitating community-guided research [37].

## 4. Community engagement from the outset

**Figure 3.**
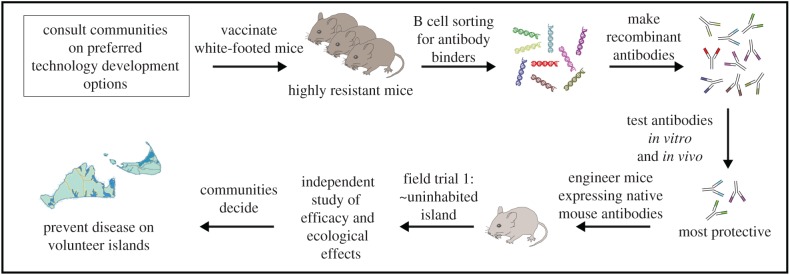
Research plan to identify native white-footed mouse antibodies conferring protection against pathogens carried by deer ticks or against deer ticks themselves, if tick feeding is in fact antibody-mediated (more testing is required to understand the mechanisms that confer resistance to tick feeding). Antibodies will be encoded in the germline via CRISPR to confer heritable resistance, and ecological effects tested by introducing engineered mice onto one or more sparsely inhabited islands prior to decisions by the communities of Nantucket and Martha's Vineyard. (Online version in colour.)

During the aforementioned meetings, we presented a variety of technical options to residents and visitors of the islands of Nantucket and Martha's Vineyard. The first set of options concerned the type of mouse immunity. Although mice are good reservoirs, because they seldom acquire sterilizing immunity to the deer tick transmitted agents like other mammals, they can be deliberately immunized. Passive immunization using anti-OspA abrogated Lyme disease reservoir competence, demonstrating that antibody alone is sufficient for this effect [38]. Thus, identifying many such antibodies and encoding them in the germline should confer heritable immunization. Antibodies targeting individual pathogens such as *B. burgdorferi* should prevent only the specific associated disease, while conferring resistance to ticks (if possible with antibodies) could block the transmission of all pathogens transmitted by deer ticks. Mice could therefore be engineered to be anti-disease, anti-tick or both (figure 3).

A key advantage of relying on antibodies is that this class of molecule is already abundant in the environment, constituting approximately 40% of total serum protein in mammals [39]. Being proteins, they are unlikely to be absorbed intact by predators and are subject to rapid microbial decomposition. As a result, using targeted antibodies to create heritable resistance should result in many fewer unwanted ecological interactions than would introducing a type of molecule not normally present in the ecosystem.

**Figure 4.**
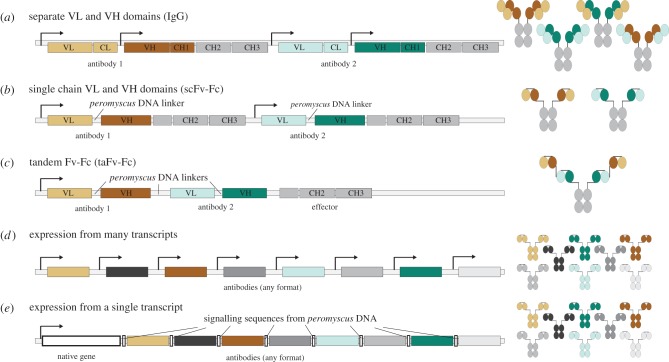
Potential designs for encoding antibodies in the germline using only native DNA from white-footed mice. (*a*) Multiple antibodies can be encoded as separate light and heavy chains, but the risk of incorrect chain assortment and reduced function rises with each antibody so encoded. (*b*) Correct antibody pairing can be ensured by linking heavy and light chains together in a single-chain format (scFv-Fc) using DNA linker sequences from white-footed mice. (*c*) Tandem scFvs can be similarly linked, potentially improving binding and reducing the required number of expression units. (*d*) The most straightforward expression method includes a separate promoter for each chain. Our current preference is to use the albumin promoter for liver-specific expression because liver cells already efficiently secrete biomolecules into serum. (*e*) Including DNA fragments from white-footed mice that when assembled produce simple molecular functions such as ribosomal skipping, which may not be found in white-footed mice, might improve expression and consequently resistance. VL, light chain variable region; VH, heavy chain region; IgG, immunoglobulin G; CL, light chain constant region; CH, heavy chain constant region.

The second set of options concerned the source and arrangement of the engineered DNA (figure 4). On one end of the spectrum, it may be possible to generate sufficient heritable resistance by exclusively incorporating native white-footed mouse DNA fragments, rearranged so as to recreate molecular functions already present in mice. The resulting organism would be cisgenic, meaning all of its DNA sequences would be derived from local populations of the same species. On the other extreme, directly incorporating known protective *Mus* and *Homo* antibodies against *B. burgdorferi* would accelerate development, using viral processing sequences would likely improve resistance, and including entire foreign genes such as CRISPR with no equivalents in white-footed mice could make introduction more efficient by incorporating a form of drive. The research team described this spectrum of options so that members of the community could form an opinion and express their preferences.

In outlining the second set of options (without input from S.W.E. and J.L.), we believed that many people are more favourably disposed towards cisgenic than transgenic engineered organisms [40], and that this likely relates to the nearly universal perception that life is a tree, implying that DNA does not move between distantly related species. As a way of addressing this incorrect assumption about genetic transfer, we often ask meeting participants who have eaten beef in the past month to raise their hands, and then note that 25% of the cow genome originated in snakes owing to an evolutionarily recent horizontal gene transfer event [41]. But we also make clear that whatever their reasoning, it is their environment, and so the decision to alter the environment should also be theirs. Moving forward, we will work to clarify this message and interpret feedback with the help of S.W.E. and J.L.

The third set of options concerns the method of introduction. Because Nantucket and Martha's Vineyard are islands, introducing sufficient engineered mice with dominant resistance should result in most descendants exhibiting resistance. However, the trait should gradually be lost over decades because it is not anticipated to improve mouse reproduction. Release could be accomplished all at once or over multiple years. Importantly, the mouse population need not exceed the normal yearly maximum at any time. For example, introducing 300 engineered mice into a field harboring a population nadir of 100 wild mice in the spring is unlikely to result in a population greater than the normal maximum of 500–800 mice in the fall owing to still-extant ecological pressures such as predation, disease, weather and limited food availability resulting in negative density dependence [42]. Nonetheless, predators and prey will be carefully monitored both before and after release to evaluate the environmental impact of introduced mice. Mouse fitness and reproductive capacity will be studied through common garden experiments with laboratory-reared and wild mice in island environments. To increase survival and promote mating, we are also experimenting with nest-boxes and other strategies. This research will inform the number of mice that are ultimately released.

As an alternative to inundative release, foreign CRISPR genes could be incorporated to create a local drive system that would confer an inheritance advantage to the resistance genes, allowing them to spread from a smaller number of released animals to a much larger population. We made it clear that we would not build a self-propagating CRISPR gene drive under any circumstances [1], as such a construct would likely spread uncontrolled to the mainland and all other populations of white-footed mice [7]. However, we might be able to construct a localized ‘daisy drive’ system to spread resistance, which would involve releasing orders of magnitude fewer mice [26]. This could be done using only DNA present within mice, but in this case the CRISPR components would be located within commensal bacteria in the mouse gut, not the mouse's own cells. We explained to those who attended our presentations that this method was still theoretical and may not prove to be possible, but that we would pursue it for their islands if requested.

We (J.B., S.R.T. and K.M.E.) presented these options at the first three meetings and then asked for an informal show of hands to express support for various options. Judging from the results and subsequent conversations, it appeared that our presentations resulted in a strong preference for immunizing mice against both Lyme disease and ticks if possible. We did not ask for a show of hands on the second set of options, but post-meeting discussions suggested a smaller majority preferred using only native DNA from white-footed mice if possible, ruling out a CRISPR-based local drive. Subsequent meetings have refined these choices in light of community suggestions and concerns while remaining broadly consistent with the apparent initial preferences. Experiments for the Mice Against Ticks project did not begin until after these initial community meetings.

### (a) Notable successes and failures of our community engagement strategy

Though a young project, Mice Against Ticks has already demonstrated areas of strength, made several mistakes, and confronted a number of open questions (table 1). Some of these may be useful for other ecotechnology development projects.

**Table 1.** Relevant successes and mistakes of Mice Against Ticks to date.

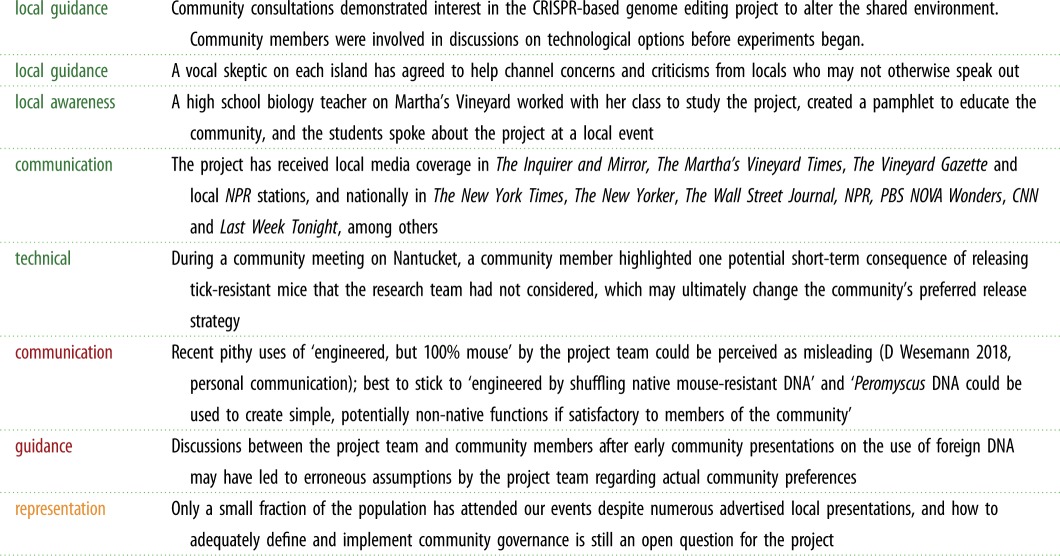

### (b) Project timeline: towards field trials

The island communities of Nantucket and Martha's Vineyard are socioeconomically and educationally diverse, with a very high average level of education suggesting they may be well-suited to guiding research. In the summers, the year-round residents are joined by groups of comparatively well-to-do summer residents and tourists, transiently increasing the local populations by a factor of 10 and including an unusually high number of prominent scientists. Both islands have long traditions of New England-specific town hall democracy. Some summer residents retire to become permanent residents, thereby obtaining the right to vote in matters of local governance. Nantucket is a single polity, while Martha's Vineyard comprises six different towns, one of which includes the separate island of Chappaquiddick. Questions of project governance are beyond the scope of this manuscript; we only note here that the desire of the team to date has been to engage with existing local institutions.

**Figure 5.**
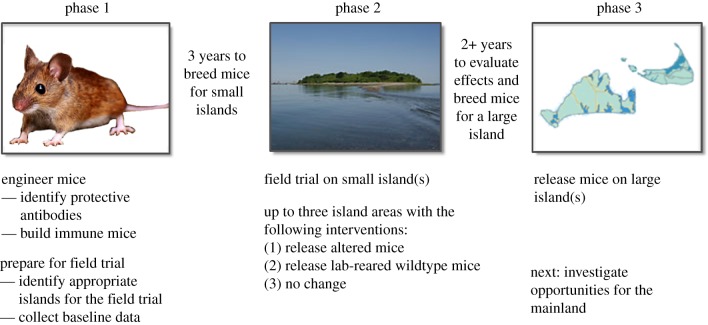
Project phases and minimum time required given the best-case scenario. (Online version in colour.)

Once the research team has generated and bred a sufficient number of heritably resistant mice, the ecological effects of the intervention could be tested in field trials on small, mostly uninhabited private islands or one large private island (figure 5), because mice are unlikely to travel between test sites. The team has already engaged with the owners of several potential field trial islands. These trials will compare the effects of releasing resistant mice with the effects of releasing an equivalent number of wild-type mice relative to a control island with no intervention. Monitoring will be informed by suggestions from community members and the results will be analysed by independent experts.

Ecological studies of candidate field trial islands are ongoing to establish appropriate comparisons to the existing wealth of data on tick-borne disease ecology on Nantucket and Martha's Vineyard (20+ years gathered by S.R.T.). If and when island(s) are deemed suitable, infrastructure can be put in place to facilitate limited field trials to observe gene flow patterns and determine optimal release methods by releasing wild-caught mice in a variety of circumstances, including providing them with biodegradable nest-boxes [43].

### (c) Molecular biology: research achievements and future directions

To isolate white-footed mouse antibodies against OspA, a *B. burgdorferi* antigen [44], we adapted a method for cloning and expressing antibodies from single isolated B cells [45] with the guidance and assistance of the Wesemann lab at Harvard Medical School. For the proof of principle, IgG1+ B cells from OspA-immunized white-footed mice from a colony derived from Martha's Vineyard in 1994 were labelled with OspA-fluorophore conjugates and sorted by fluorescence-activated cell sorting. A single-cell RT-PCR strategy was employed to amplify Ig heavy and light chain variable region gene transcripts. Candidate anti-OspA antibodies are being tested for binding affinity and borreliacidal activity *in vitro* and within the infectious tick, and will be followed by epitope mapping. Assuming tick resistance is antibody mediated [46,47], candidate anti-tick antibodies will also be tested for antigen binding affinity and undergo target epitope binding studies. Each antibody will be purified and injected into white-footed mice, which will be challenged with infected ticks to determine the extent of *B. burgdorferi* clearance or tick rejection relative to control antibodies.

Multiple antibodies may be expressed from the same cell type in a variety of formats using solely white-footed mouse DNA (figure 4), though some of these options require the rearrangement of native DNA fragments to achieve simple functions such as ribosome skipping that may not normally be found in white-footed mice. Current technical plans involve harnessing the albumin enhancer-promoter [48]. For each design option, efficacy and phenotypic data will be presented to members of the community and their representatives to help determine the final version to be tested in field trials (figure 5).

Because heritable genome editing has not yet been achieved in white-footed mice, we will test a variety of delivery methods for CRISPR-based insertion, including embryo injection and i-GONAD [49]. We will measure the extent of resistance in edited offspring, and if judged sufficient, begin separate outcrossing to captured wild *P. leucopus fusus* (an insular endemic of Martha's Vineyard) and to *P. leucopus noveboracensis* (found on Martha's Vineyard, Cuttyhunk and Nantucket) while preserving the introduced resistance alleles by genotyping.

### (d) Possible expansion to the mainland

In principle, the same antibody-encoding genes used to confer resistance on the islands could be efficiently introduced in a mainland town using a CRISPR-based daisy threshold technology to keep the engineered genes within its borders [50]. Early laboratory research on daisy threshold systems in other species of rodents is underway in the Esvelt lab in consultation with Maori iwis and NGOs in Aotearoa, New Zealand. If successful in *Mus*, one or more mainland communities may be approached to consider whether and how to pursue research on daisy threshold for the heritable immunization of white-footed mice, potentially including another island field trial.

Mice Against Ticks may also be considered a pilot project or proof of principle for ecological engineering intended to remove the animal reservoirs of other zoonoses. The methods used to heritably immunize white-footed mice could be extended to the rodent reservoirs of hantaviruses (hantavirus pulmonary syndrome, haemorrhagic fever with renal syndrome) or arenaviruses such as those causing Lassa fever or Bolivian haemorrhagic fever.

## 5. Discussion

When developing a new technology, it is often best to begin by working on what, at least initially, may be considered a simple, safe and straightforward application. Mice Against Ticks aims to develop an new way of preventing tick-borne disease that appears to meet these criteria. Heritably immunizing the white-footed mice thought to be responsible for infecting more ticks than any other species by harnessing naturally occurring resistance may be the simplest long-lasting intervention that may be technically and socially feasible. Mice Against Ticks is starting small by working with members of island communities to identify a suitable alteration capable of delivering nearer-term benefits without the added complexity of CRISPR-based gene drive, which might be developed and combined with those same alterations later on for other island or mainland adoption.

Mice Against Ticks is also a very favourably situated project with respect to the goal of developing a new model of community-guided science, although this aspect of the project is still in its early stages of development. Numerous public health and development projects have employed community-directed initiatives [51], and at least one earlier ecological engineering project consulted communities regarding safety testing [52]; our model is distinct in featuring consultation at the earliest phases of biotechnology development. The high level of local expertise available to Nantucket and Martha's Vineyard is virtually unmatched; many community members are likely to know someone with the technical ability to evaluate at least some of the scientific details of the project. Combined with their long tradition of town hall democracy, the islands appear to be highly favourable environments in which to experiment with feedback loops between communities and research design. Once developed and tested, these strategies might be compared with others and implemented in progressively more challenging environments to determine the limits of community-guided technology development [53].

Writ large, Mice Against Ticks is an effort to shift research norms towards greater consideration of consequences at a much earlier stage of technology development.

## Data accessibility

This article has no additional data.
